# Quantifying the influence of optical coherence tomography beam tilt in each retinal layer

**DOI:** 10.1371/journal.pone.0325217

**Published:** 2025-06-10

**Authors:** David Bissig, Shasha Gao, Haohua Qian

**Affiliations:** 1 Department of Neurology, University of California – Davis, Sacramento, California, United States of America; 2 Department of Ophthalmology, the First Affiliated Hospital of Zhengzhou University, Zhengzhou, China; 3 Visual Function Core, National Eye Institute, National Institutes of Health, Bethesda, Maryland, United States of America; Save Sight Institute, AUSTRALIA

## Abstract

**Purpose:**

Well-aligned microstructures within the retina – like retinal nerve fiber layer (RNFL) axons – differentially reflect light depending on its angle. Our goal was to quantify the influence of optical coherence tomography (OCT) beam tilt on reflectivity of each layer of the mouse retina.

**Methods:**

We collected OCT images in a single plane capturing the optic nerve head, temporal retina, and nasal retina, while tilting the OCT beam at various angles. We converted signal intensities to estimated attenuation coefficients (eAC). The attenuation coefficient describes how quickly the remainder of an OCT beam’s light is absorbed or scattered at a given depth into the retina. A single-ellipse model based on prior literature was calculated at each retinal depth, describing the maximum eAC across all tilts (ellipse semi-major axis), the beam tilt eliciting that maximum eAC, and eAC’s dependence on beam tilt (semi-major versus semi-minor axes). *Post hoc*, the inner retina bore an unexpectedly complex relationship between beam tilt and eAC, which we explored with a two-ellipse model.

**Results:**

eACs in the temporal and nasal retina were dissimilar at specific beam tilts, but this was near-completely explained by differences in microstructure alignment. Dependence on beam tilt was substantial over the photoreceptors, but non-zero in all retinal layers. *Post-hoc*, two-ellipse models implied that microstructures vitread to the external limiting membrane were well-aligned with the photoreceptor inner and outer segments, and a small fraction (≥0.3%) of that tissue is especially translucent.

**Conclusion:**

We mapped microstructure alignment throughout the retina. Expected findings at the photoreceptor inner and outer segments are complemented by new evidence of unusually translucent microstructures spanning much of the retina, possibly representing Müller glia.

## 1. Introduction

Each layer of the retina uniquely reflects infrared light, accounting for layer-to-layer contrast in optical coherence tomography (OCT). In regular clinical practice, the operator is encouraged to direct the OCT beam perpendicular to the surface of the retina [1]. Departing from this practice creates an undulating circular scan [2], a tilted linear B-scan [1,3], and alters the layer-to-layer contrast between retinal layers. For example, reflectivities in the retinal nerve fiber layer (RNFL) and the adjacent ganglion cell layer (GCL) are differentially sensitive to beam tilt [4]. Placement of the RNFL/GCL border is thereby influenced by beam tilt, leading to unwanted variance in RNFL thickness measurements [2]. The same effect is sometimes advantageous: For instance, Lujan et al. [3,5] deliberately tilted the OCT beam – a technique described as directional OCT (D-OCT) – to maximize the visibility of the Henle fiber layer, improving structural measurements.

Why is reflectivity more tilt-dependent in some layers of the retina than others? In the RNFL, ganglion cell axons are stacked in parallel, and scatter light in the same way as idealized stacked cylindrical structures [6]. Pertinent only to species with a macula, the same explanation is offered for the Henle fiber layer [3]. The OCT appearance of photoreceptor inner and outer segments is highly tilt-dependent [7,8], and this is attributed to the parallel alignment of photoreceptors as well as their waveguiding properties (accounting for the Stiles–Crawford effect). Reflectivities in the other major layers – the GCL, the inner plexiform layer (IPL), inner nuclear layer (INL), outer plexiform layer (OPL), outer nuclear layer (ONL), and the retinal pigment epithelium (RPE) – are generally presumed to be so insensitive to beam tilt that they serve as reference tissues for signal normalization [3,4,7,9]. The ONL is mostly occupied by spherical nuclei, accounting for the prediction that measured reflectivity would not be affected by beam tilt [9], and the same could be argued for the INL and GCL. In the RPE, melanin dominates reflectivity, and this layer does not appear especially sensitive to beam tilt [9]. Reflectivity might be direction-sensitive for individual microstructures within the IPL and OPL, but because those microstructures are not especially well-aligned, a “bulk” measurement for the IPL or OPL might be insensitive to tilt [7].

Neither the reflectivity of a layer, nor the tilt-dependence of that reflectivity, are immutable properties. Instead, they may change with modifications in the microstructures – or their parallel alignments. This is most obvious in large insults, like central retinal artery occlusion [10], but more subtle reflectivity changes have been documented with D-OCT *(i)* in the RNFL of glaucomatous eyes, where reflectivity measurements augment disease detection compared to structural measurements alone [11] and *(ii)* in photoreceptor layers of those afflicted with age-related macular degeneration, where the tilt-dependence of reflectivity is altered, implying that cones over drusen are not merely misaligned, but that their optical properties are also abnormal [12].

To advance the field of D-OCT, we provide a large dataset from the central retina of healthy control mice (doi.org/10.5061/dryad.95x69p8th). In selecting a region of the retina to image, we were inspired by Lujan et al [3]. They imaged the human fovea, where known mirror-opposite tilts in Henle fiber alignment (nasal versus temporal parafovea) permitted an excellent regional contrast within a single B-scan. Mice lack a fovea, but we know that the photoreceptor inner and outer segments are angled in the central-most retina [13], and their angle in the temporal retina is roughly the mirror reverse of that seen in the nasal retina. We therefore imaged the central temporal and nasal retina, to test whether well-modeled D-OCT data reports on that photoreceptor tilt. Beyond the photoreceptors, we recognized that it is difficult to plan D-OCT studies of other retinal layers without normative data: Are most layers actually insensitive to beam tilt in a healthy retina? If they are affected by beam tilt, what is a good mathematical description of the impact of tilt? We therefore collected enough data to support *post-hoc* modelling of D-OCT for each retinal layer.

## 2. Materials and methods

### 2.1. Animals and image acquisition

All procedures involving animals were conducted under an approved animal care protocol by the National Eye Institute Animal Care and Use Committee and in accordance with ARVO guidelines on the humane use of animals in ophthalmic and vision research. 15 adult wild-type C57 mice (Jackson Labs, Bar Harbor, ME, age ~ 3 mo, *ad libitium*) of either sex was used in this study. The mice were kept in regular animal housing under a 50 lux 14:10 hour light/dark cycle.

**Fig 1:**
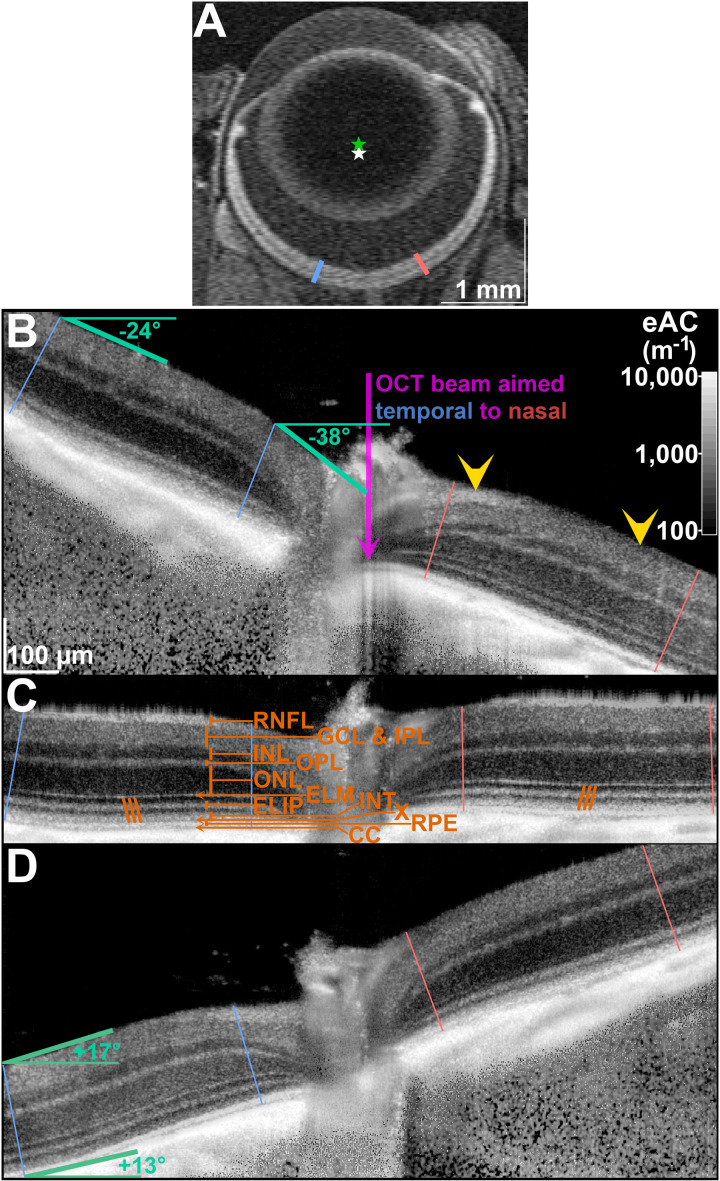
Qualitatively, beam tilt has a layer-specific influence on retinal eAC. A: To illustrate the anatomic location of OCT images, we here provide a T_1_-weighted magnetic resonance image of a mouse eye (axial resolution 21.875 μm; collected as part of a study [14] in Dr. Bruce Berkowitz’s lab). Blue and red marks are placed in the retina ±625 µm from the optic nerve head, measured along the retina-choroid border. The contour of that border approximates a circle whose geometric center is marked with a white star, and is very near the optical rear nodal point for the whole eye (green star; ~ 1.62 mm interior to the corneal surface [15] for a mouse with an axial length of ~3.35 mm). B-D: Estimated attenuation coefficient (eAC) OCT images of the same retina, captured at different beam tilts. Retinal data were sampled 175−625 µm (measured along CC) from the center of the optic nerve head in both the temporal (figure left; blue lines) and nasal (figure right; red lines) retina. The path of the OCT beam is vertical (purple arrow). Retinal layers are marked in panel C; retinal nerve fiber layer (RNFL), ganglion cell layer and inner plexiform layer (GCL & IPL), inner nuclear layer (INL), outer plexiform layer (OPL), outer nuclear layer (ONL), external limiting membrane (ELM), the “ellipsoid zone” (ELIP), the outer segment tips at the “interdigitation layer” (INT), the retinal pigment epithelium (RPE), and choriocapillaris (CC). The anatomical correlate of a hyporeflecting band “X” between INT and RPE is controversial [16]. B: The retina appears rotated clockwise because the OCT beam entered the pupil temporal to the optical axis of the eye. Path length to the temporal retina is reduced – it appears closer to the camera and higher in the image – compared to the path length to the nasal retina. Equivalently, the beam is tilted relative to anatomical borders, and the magnitude of beam tilt is derived from the apparent angle of tissue borders. By convention [4], negative angles indicate an apparent clockwise rotation of the retina, and therefore a beam that is aimed in the temporal-to-nasal direction. Prior efforts [3,4] assigned a single angle measurement per OCT image, but this was inadequate for the current dataset, as exemplified by variation at the temporal retina’s RNFL (−38° at 175 µm from the optic nerve, and −24° at 625 µm), and by the variation in RNFL signal within the nasal retina: When the retina-vitreous border is angled < −20° (right yellow arrowhead) the RNFL is darker than the adjacent retinal layers. At less-severe tilts (closer to 0°; left yellow arrowhead) the RNFL is brighter than the GCL & IPL. Note that ELIP and INT in the temporal retina is more reflective (higher eAC) than in the nasal retina. This pattern is also present in group-average data (Fig 2, top), and changes as beam tilt varies. C: When beam tilt is near-zero, the nasal and temporal retina look similar. Still, much of the outer retina (including ELIP and INT) appears slightly *less* reflective (darker grayscale; lower eAC) in the temporal retina compared to the nasal retina. Orange angled marks (\\\ and///) depict the ~ 14° tilt of photoreceptor inner and outer segments expected in the central retina [13]. D: The OCT beam is aimed nasal-to-temporal, causing an apparent counter-clockwise rotation of the retina (beam tilt > 0°). Now, temporal/nasal differences in ELIP and INT reflectivity are reversed compared to panel B, presumably because the long axes of nasal photoreceptors are now well-aligned with the OCT beam. Lines perpendicular to CC are used to digitally linearize the retina for later processing. Along those lines – like the left-most blue line – beam tilt at the retina-vitreous border (+17°) need not match the beam tilt measured at CC (+13°). We use measurements at those two locations to assign a unique beam tilt to each location in the retina: Along that far left blue line, for instance, at depths one quarter, one half, and three-quarters into the retina, the assigned tilts are respectively +16°, + 15°, and +14°.

OCT images were captured using an Envisu UHR2200 system (center wavelength 870 nm) (Bioptigen, Durham, NC). Mice were anesthetized with ketamine (100 mg/kg) and xylazine (6 mg/kg), and the eye was positioned with the optic nerve head in the center of the OCT scan. Averaged B-scans (40x, 1000 A-scan for 1.4 mm) were first captured with the beam centered for the eye (i.e., creating a level OCT image as in Fig 1C). B-scan OCT images with different amounts of tilt were captured by manually moving the mouse eye horizontally for an arbitrary distance in both directions. Those distances varied from eye to eye, because they were intermediate steps between the first acquisition, and the most extreme tilts we could obtain for a specific eye. We excluded images wherein wrap-around artifact or low signal (if outer nuclear layer and vitreous within two standard deviations of one-another) contaminated more than half of our nasal or temporal regions of interest. After exclusions, we analyzed n = 27 eyes from the fifteen mice. For completeness, the data repository (doi.org/10.5061/dryad.95x69p8th) retains individual images that were excluded from an otherwise successful imaging session.

### 2.2. Image processing

Clinically, it is common to view OCT images with log-transformed pixel values, which accommodate the wide dynamic range of retina layer reflectivities, at the expense of slightly shifting anatomical borders [17]. This default output from our Bioptigen system was mathematically transformed into pixel values directly (linearly) proportional to the power of the signal measured by the OCT device, which are used to calculate estimated attenuation coefficients (eAC) based on Vermeer et al [18]. At a given distance from the OCT device, *z*, these pixel values are denoted *U(z)*. The top half of each image was occupied by vitreous, where – being transparent to allow the passage of light – tissue reflectivity is so low that *U(z)* approximates noise, *N*. In each set of (≥ 9) images per eye, those noise values (*N(z)*) followed a gaussian distribution. In the area occupied by the vitreous, both distribution parameters (mean, σ) increased linearly with distance from the OCT device (more noise at greater *z*; the mean/σ ratio was nearly constant). This trend was extrapolated into the image region occupied by the retina, and we used the “rnorm” function in R v4.0.5 to sample from those distributions to estimate values for *N(z)*, constrained such that *U(z)* – *N(z)* ≥ 0.

Vermeer et al [18] offer another step, dividing the noise-subtracted image (*U(z)*-*N(z)*) by a signal decay factor to correct a fairly small source of variance – in their system, a 3 dB signal loss after a 4*.*7 mm shift in distance from the OCT camera. For our commercial system, shifts in distance between images (and with different beam angles) are generally < 0.4 mm, and we anticipated that no correction for signal decay would be needed. Exploration of z-position’s impact on our noise-subtracted signals was supportive (not shown). Thus, we modify Vermeer et al’s calculations to ignore this signal decay factor, so that *I(z)*=*U(z)-N(z)*, and calculate attenuation coefficients one A-scan at a time using the seventeenth equation from that manuscript [18]. In deference to this departure, and other attenuation coefficient calculation strategies [19], we refer to our results as *estimated* attenuation coefficients (eAC). eAC is reported in m^-1^ and log-transformed, with log_10_(eAC)≈0 representing a tissue too transparent to distinguish from background noise.

In ImageJ, images were resized in the axial direction from the original 1.4 µm × 1.53 µm per pixel to a nominal image resolution of 1.4 µm × 1.4 µm per pixel. Using custom scripts in R and manual labeling, the retina-vitreous border and the choriocapillaris (CC) were identified. Smooth spline functions allowed for easy sampling of the retina every 1 µm along the contour of CC. The full depth of the retina was sampled every 1 µm along perpendiculars radiating from CC, and organized as a spatially linearized image, like a cross-section of a flat-mounted retina. The slopes of the smooth spline along the CC, and of another smooth spline along the retina-vitreous border, provided pixel-by-pixel estimates of beam tilt throughout the retina (Fig 1).

To facilitate comparisons across animals, data were mapped on to a common spatial template as in previous work [4]: We located the border between the vitreous and RNFL (alongside the inner limiting membrane), the RNFL/GCL border, the IPL/INL border, the OPL/ONL border, the ELM, and the hyporeflective band just exterior to the retinal pigment epithelium (RPE) and Bruch’s membrane, which is attributed to the CC [20]. Next, the image was dynamically resampled between each of these anatomical landmarks. For instance, at each distance from the optic nerve (along the CC), ten equally spaced points were sampled between the vitreous/RNFL and RNFL/GCL borders: Where the RNFL is thick, these ten points are spaced proportionally farther apart (in μm) than where the RNFL is thin. The sampling strategy approximates the average appearance of the retina in our region of interest – 175–625 μm nasal and temporal to the optic nerve – when sampled every ~1 μm into the depth of the retina. In total, 200 points were sampled between the retina/vitreous border and the CC, and mapped onto a scale of %Depth.

### 2.3. Planned modelling of beam tilt’s influence on eAC: Averaged data

**Fig 2:**
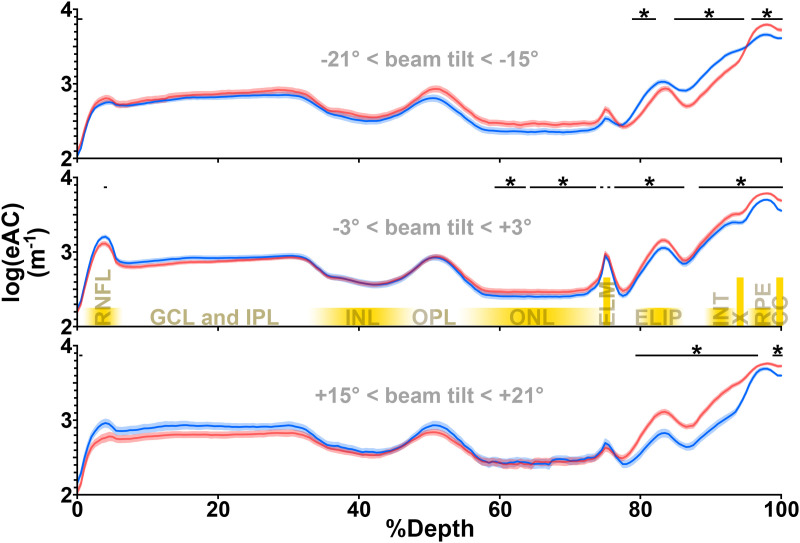
Quantitatively, beam tilt has a layer-specific influence on retinal eAC. Mean (±s.e.m.) eACs from the mouse retina are aggregated at different beam tilts. In all three panels, a black band is used to denote differences (q < 0.05) between the temporal (blue) and nasal (red) retina. To avoid visual clutter, * is marked only when three or more contiguous %Depths show significant differences. The photoreceptor inner and outer segments (roughly corresponding to the ELIP and INT layers, respectively) consistently show significant differences between the temporal and nasal retina. There, eAC is higher in the temporal retina at negative beam tilts, matching photoreceptor alignment [13] and patterns seen in individual retinas (Fig 1B). The pattern reverses at positive beam tilts (figure bottom; corresponding to Fig 1D). The opposite trend (nasal>temporal at negative tilts, temporal>nasal at positive tilts) seems present through much of the inner retina, but is better-captured in later analyses. Even when beam tilt is ~ 0° (middle panel), eACs in the temporal and nasal retina are significantly different at CC, RPE, INT, the faint relatively hyporeflective band (“X”) between INT and RPE, as well as ELIP, the ELM, much of the ONL, and a tiny portion of the RNFL.

We first analyzed averaged data from all 27 eyes to visualize and trend beam tilt’s influence on eAC in each retinal layer. For each B-scan, pixel values at a given %Depth into the retina report on the eAC over a modest range of beam tilts, varying a few degrees with the contour of the retina (Fig 1). Because multiple B-scans (≥ 9) were obtained from each eye while deliberately altering the beam tilt, a wide range of tilts are interrogated for each eye. These departures from the normal perpendicular angle between the beam and the retina are reported with a sign convention as in Fig 1. Each B-scan image was divided into temporal and nasal hemiretinas, which were analyzed separately. We binned data according to beam tilt, and calculated the median log(eAC) value for each bin. Because the data are nested (most mice contributed two eyes), generalized estimating equations (GEE) were used to calculate the mean and standard error of those values. A bin size of 1° is used except for Fig 2 where a large (6°) bin size illustrates broad temporal versus nasal trends. The next step was applied to beam tilts wherein at least one third of the sample (nine eyes) contributed data.

Two approaches were used to model log(eAC) as a function of beam tilt. The first is based on Meleppat et al, [9] for whom a gaussian function provided a reasonable fit to data from the RNFL, the external limiting membrane (ELM), the photoreceptor inner–outer segment junction (here labeled as the ellipsoid zone (ELIP)), and the RPE. With the Levenberg-Marquardt algorithm implemented in R’s minpack.lm library, we fit the four parameters in Equation 1: a_0_ represents non-directional attenuation – the lowest eAC possible of a structure, regardless of beam tilt – which we obliged to be no lower than our detection limit (log(eAC) > 0). The peak intensity and directionality are respectively a_1_ and ρ, while x is the beam tilt in the horizontal (temporal↔nasal) direction, which is compared to the beam tilt with the greatest eAC (x_0_). Because our changes in beam angle were on the horizontal meridian (temporal↔nasal), the suggested [9] vertical (y-y_0_) component was expected to be ~ 0, and does not appear in Equation 1.


$$eAC=a_{0}+\;a_{1}\times 10^{-\rho {\left(x-x_{0}\right)}^{2}}$$ (1)

**Fig 3:**
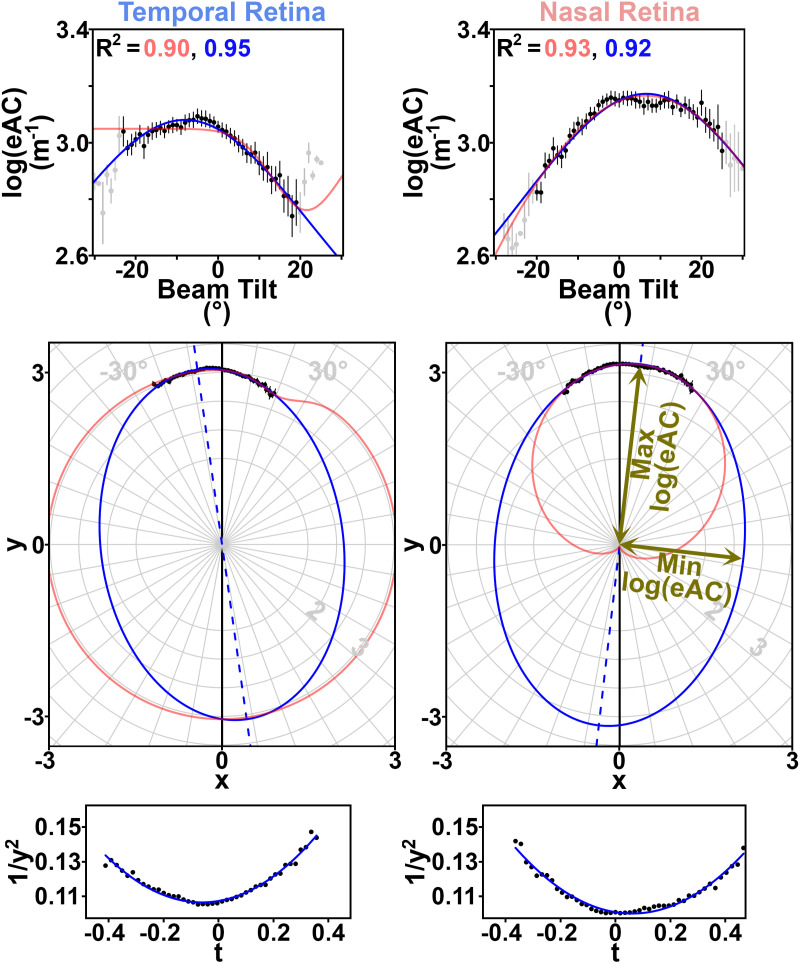
Binned group-average data from the ellipsoid zone (83.5%Depth) illustrate estimated attenuation coefficient (eAC) variation according to beam tilt. The temporal (figure left) and nasal (figure right) retina are analyzed separately. Top: Group mean (±s.e.m.) log-transformed eACs as a function of beam tilt, formatted like in prior work [9]. Black points are used when at least nine eyes contribute to that mean; only those points are used for single-ellipse (blue) and gaussian (red) fits and their accompanying multiple R^2^ values, which are calculated with without additional consideration/weighting of the variance at each point. Data from fewer eyes were available at extreme beam tilts (gray points). The peak eAC is at negative beam tilts in the temporal retina, indicating that this part of the retina is most reflective when the OCT beam starts temporal to the optic nerve head, and is aimed nasally (as in Fig 1B). Middle: Data are re-plotted in polar coordinates (“*x vs y space*”) where tilt and log(eAC) are demarked with the gray radial grid, the model ellipse is centered at (x,y)=(0,0), and the lower half of these plots (where y < 0) describes predictions for |tilt| > 90°. For the nasal retina, the gaussian fit (red) predicts a transparent ellipsoid zone (log(eAC)≈0) for a tilt of ~180° – if the OCT beam first passed through the sclera – in conflict with prior work [21]. The minimum and maximum eACs (as plotted in Fig 4) respectively are the semi-minor axis and the semi-major axis of a best-fit ellipse. This polar view highlights the orientation of the major reflectors in this part of the retina (dashed blue line; tilted like the orange hash marks in Fig 1C). Bottom: The data are re-plotted in “*t vs 1/y*^*2*^ *space*”, where t = x/y, and each point’s x and y values are captured from the middle plots. A quadratic fit in this space (Equation 2) transforms into an ellipse centered at (x,y)=(0,0) in the middle plots.


This approach from Meleppat et al [9] *(i)* is not compared to another model in their study – perhaps one with fewer variables would spare statistical degrees of freedom, *(ii)* its applicability to other layers (e.g., IPL) is not discussed, and *(iii)* prior literature implies that a gaussian fit is inadequate to describe the entire relationship – spanning all 360° – between beam tilt and signal intensity: When the OCT beam is aimed through the sclera (a tilt of 180°) the ELM is about as reflective as ELIP, and is more reflective than the ONL [21]. Meleppat et al [9] found that ELM signal rapidly diminishes as the OCT beam is tilted off-perpendicular, becoming hard to distinguish from the ONL even at tilts of ±15°. A gaussian fit offers no chance for ELM signal to rebound around 180°. A simpler and cyclical model was therefore favored in our second approach: Inspired by the mathematic treatment of diffusion MRI data, which uses a three-dimensional ellipsoid, we fit a single ellipse to our two-dimensional OCT data: The data were plotted in polar coordinates overlaid on a cartesian *“x vs y space*” (middle of Fig 3). In the special case of an ellipse centered on the origin – as desired for our models – this can be transformed to “*t vs 1/y*^*2*^ *space*” (bottom of Fig 3) via Equation 2.

$$at^{2}+bt+c=\frac{1}{y^{2}}\;,\;where\;t=x/y$$ (2)

In the equation for a parabola, Equation 2, an ordinary least-squares quadratic best-fit in *t vs 1/y*^*2*^ *space* yields estimates for coefficients a, b, and c. The best-fit line is transformed back to other spaces as needed.

The three-parameter ellipse model (either a, b, and c as described above, or in *“x vs y space”* its angle of orientation along with the lengths of its semi-major and semi-minor axes) is not nested within the four-parameter gaussian model. We therefore compared the Aikake Information Criterion (AIC) calculated in “*tilt vs log(eAC) space”* from each model’s fit of the data at each %Depth. In this search for the better model to use throughout the depth of the retina, a superior model would have lower AICs at the preponderance of retinal %Depths.

### 2.4. Planned modelling of beam tilt’s influence on eAC: Estimates from individual hemiretinas

We next characterized beam tilt’s influence on individual hemiretinas. At each %Depth: ***(i)*** log(eAC) and beam tilt were extracted from each pixel (~450) of each OCT image (≥ 9). This generates ≥ 4050 log(eAC) values per hemiretina at that %Depth. ***(ii)*** any log(eAC) values < 0 were censored, and ***(iii)*** univariate outliers (|z-score| > 3) were censored. The relationship of *tilt vs log(eAC)* was then characterized using GEE with an autoregressive relationship between adjacent pixels within each B-scan.

The literature-based expectation (see Introduction section) was either that log(eAC) would have no relationship to tilt – as previously assumed for the ONL [9] – or that it would have a curvilinear relationship with a single peak, justifying an elliptical (or gaussian) fit. Hypothetically, if the peak eAC were not captured in the range of tilts tested, then only the rise or fall of the curvilinear relationship would be visible, and the relationship between tilt and log(eAC) might look linear. To cover all three possibilities, we ***(iv)*** calculated a linear *tilt vs log(eAC)* fit, and compared it to a quadratic fit. ***(a)*** If the quadratic was statistically (p < 0.05) superior to the linear fit, then values were transformed into *x vs y space* (Fig 3, middle) and *t vs 1/y*^*2*^ *space* (Fig 3, bottom) where we fit a quadratic GEE based on Equation 2. Ellipse features (e.g., semi-major axis) were logged when that best-fit line was transformed to *x vs y space*. In contrast, ***(b)*** when log(eAC) varied linearly with beam tilt, this implied that the best-fit ellipse was angled by roughly ±30–60° in *x vs y space*, because neither the peak (vertex) nor trough (co-vertex) eAC was captured in the broad range of beam angles tested. In this case, the linear best-fit for *tilt vs log(eAC)* was transformed into *x vs y space*, where we fit an ellipse using R’s conicfit library. Finally, ***(c)*** if there was neither a linear nor a quadratic relationship (p > 0.05) for *tilt vs log(eAC)*, or the tentative relationship did not hold once values were transformed, then the ellipse assigned in *x vs y space* was a circle with a radius of mean log(eAC), and no ellipse angle of orientation was calculated.

The above steps pursue literature expectations in a statistically robust manner: To our knowledge, there is no direct GEE (nor multi-level modelling) method of fitting nested observations with an ellipse. We accomplish that goal indirectly by transforming to *t vs 1/y*^*2*^ *space* and fitting a quadratic function. *Post hoc,* we uncovered complex *tilt vs log(eAC)* relationships that are missed by step ***iv***. The INL is illustrative (see below), where the *tilt vs log(eAC)* relationship is neither linear nor quadratic (nor gaussian, nor fit well with the single-ellipse model).

### 2.5 Planned statistics for estimates from individual hemiretinas

For each hemiretina, for each eye, at each %Depth, the single-ellipse model generated estimates of the maximum log(eAC) (ellipse semi-major axis), the minimum log(eAC) (ellipse semi-minor axis), and – if the semi-major axis and semi-minor axis were non-identical – the beam tilt at which the tissue would be most reflective. If each animal contributed only one eye, then to test whether the optimal beam tilt was non-zero (as for photoreceptor outer segments), a one-sample t-test would be used on values from the nasal retina, and another on values from the temporal retina. Because most animals contributed two eyes, the GEE equivalent is used. To compare the nasal and temporal retina, the differences (temporal-nasal) are calculated, and compared to zero with the GEE equivalent of a one-sample t-test. When a value is missing from a hemiretina (e.g., no ellipse angle of orientation could be calculated because the best-fit ellipse was a circle) we conservatively censor that retina for that statistical comparison. Because a given question (e.g., “Is the tilt for a maximum log(eAC) in the nasal retina the same as in the temporal retina?”) is repeated at each %Depth (0–100%, inclusive, in 0.5% steps), a false discovery rate threshold (q = 0.05 for 201 tests) controlled type 1 error.

To provide a convenient description of how tilt-sensitive the OCT signal is at a given %Depth, we calculate the third eccentricity of the ellipse (e″), based on the lengths of the semi-major axis (a) and semi-minor axis (b), using Equation 3:


$$e^{\prime \prime }=\;\frac{\sqrt{\left(a^{2}-b^{2}\right)}}{\sqrt{\left(a^{2}+b^{2}\right)}}\;$$ (3)

The third eccentricity was selected instead of the first eccentricity (which has the same numerator, but the denominator is simply the length of the semi-major axis) due to the resemblance to fractional anisotropy, which would be used for a three-dimensional dataset.

## 3. Results

### 3.1. Planned modelling of beam tilt’s influence on eAC: Averaged data

The relationship between tilt and log(eAC) differs in the temporal and nasal retina (Fig 2): At the temporal retina photoreceptors (ELIP, INT), eAC is highest at *negative* tilts. In the nasal retina, photoreceptor eAC is highest at *positive* tilts. More vitread layers of the retina trend in the opposite direction. Modelling at each %Depth clarified these trends.

The gaussian model (Equation 1) has previously been applied to the RNFL (1–5%Depth), the ELM (75%Depth) and the structures exterior to the ELM (76–100%Depth) [9]. There, in aggregate, we find that the single-ellipse and gaussian model are both adequate: The single-ellipse was superior (lower AIC) to the gaussian model at roughly *half* of those %Depths; 3/18 locations in the RNFL (nine from each hemiretina: 1–5%Depth, inclusive, in 0.5%Depth increments), 57/98 locations exterior to the ELM (76–100%Depth), 1/2 at the ELM (S6 Fig), and 2/4 immediately adjacent to the ELM (at 74.5% and 75.5%, respectively).

Both models underperformed for the GCL through the ONL, where *tilt vs log(eAC)* often was neither linear, nor curvilinear with a single peak. This is evident in group average data (section 3.3), but also emerges in analyses of individual hemiretinas (next section).

### 3.2. Planned modelling of beam tilt’s influence on eAC: Estimates from individual hemiretinas

**Fig 4:**
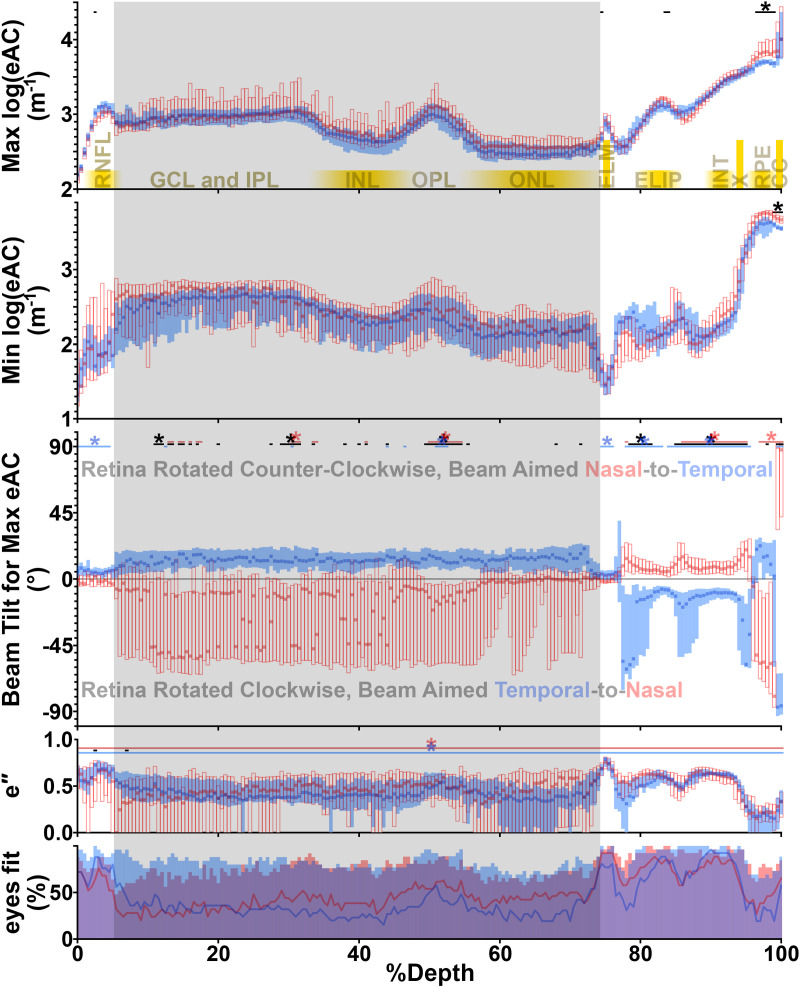
Results of the single-ellipse model at all retinal layers. In the top four panels, median (with upper and lower quartiles) data are displayed for the temporal (blue) and nasal (red) retina. A black band is used to denote temporal/nasal differences (q < 0.05). In the third (“beam tilt”) and fourth (eccentricity; “e″”) panels, we tested whether temporal and nasal data were different from 0, and color-coded bands likewise denote significance (q < 0.05). To avoid visual clutter, * is marked only if three or more contiguous %Depths show a significant difference. Through most of the *neural* retina, the maximum log(eAC) in the temporal and nasal retina is similar (excepting %Depths of 73.5, 83.5, and 84.0). Differences at the RPE are attributable to temporal/nasal differences in melanin content [22]. Throughout the *neural* retina, the minimum log(eAC) was no different in the temporal versus nasal retina (q > 0.05), and only sparse temporal/nasal differences were found in e″ (2.5 and 7.0%Depth). In contrast to the widespread temporal/nasal differences in Fig 2, this argues that the nasal and temporal *neural* retina are similar in composition, differing mainly in the orientation of the reflective microstructures within each layer. Of note, band “X” has the same high sensitivity to beam tilt as the photoreceptors – best-matching the region between ELIP and INT – arguing that “X” is a continuation of the photoreceptors. Tilt-dependence was unexpected for much of the inner retina, and inspired a further review of the single-ellipse model: The gray band spanning all plots from the GLC through the ONL highlights where a two-ellipse model is thought superior, based on *post-hoc* analyses. Fig 6 helps to explain the high variance in that span. The bottom panel shows the % of individual hemiretinas (temporal = blue, nasal = red) with a significant quadratic (solid lines) or linear relationship for *tilt vs log(eAC)* (filled transparent bars show the % of hemiretinas with a significant linear *and/or* quadratic fit). Where most eyes lacked a quadratic fit, there was not a conspicuous single peak for *tilt vs log(eAC)*, and the single-ellipse model underperformed.

Single-ellipse estimates of the maximum log(eAC), minimum log(eAC), and beam tilt of greatest reflectivity are plotted at each %Depth in Fig 4. The same figure also shows ***(i)*** the ellipse eccentricity (e″), and ***(ii)*** how often (% of hemiretinas) *tilt vs log(eAC)* was linear or quadratic. That fit was often linear – at least according to our initial analysis approach – at ~7 through ~74%Depth into the retina. An absence of curvature makes it challenging to fit an ellipse, sometimes yielding an unrealistically high estimate of maximum log(eAC), skewing the distribution of values, informing visualization of group median and quartile values in Fig 4. A better model – neither gaussian nor single-ellipse – was needed for the GCL through the ONL.

### 3.3. Post-Hoc description of beam tilt’s influence on control group eAC: The two-ellipse model

At representative locations in the RNFL (4%Depth; S1 Fig), the ELM (75%Depth; S6 Fig), the ellipsoid zone (“ELIP”, 83.5%Depth; Fig 3), the interdigitation zone (“INT”, 92%Depth; S7 Fig), one or both hemiretinas displayed the expected [9] contour on plots of beam *tilt vs log(eAC)*: As in Fig 3, there was a curvilinear relationship for *tilt vs log(eAC)* with a single peak (or broad plateau). The relationship was subtle but also present at the RPE (97%Depth; S8 Fig). While increasing model degrees of freedom would surely improve the fit to available data, the single-ellipse model was felt to adequately report on the orientation of reflective microstructures within the tissue, and log(eAC)’s relative dependence on beam tilt (non-zero e″; Fig 4).

**Fig 5:**
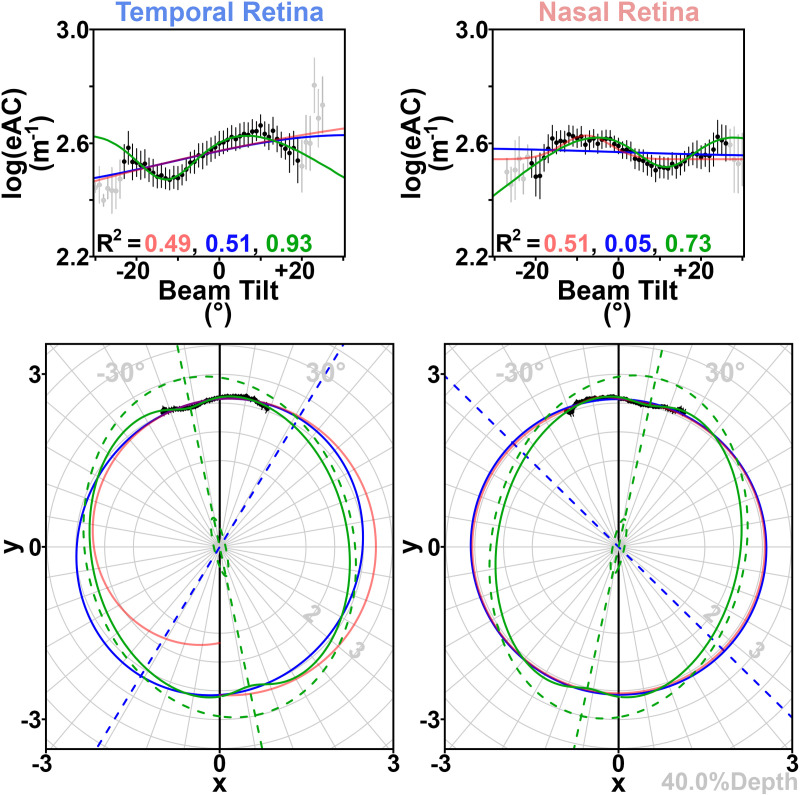
Binned group-average data from the inner nuclear layer (40.0%Depth) illustrate log(eAC) variation according to beam tilt, and the value of the two-ellipse model. The temporal (figure left) and nasal (figure right) retina are analyzed separately. Top: Group mean (±s.e.m.) log(eAC)s as a function of beam tilt, formatted like in prior work [9]. Black points are used when at least nine eyes contribute to that mean, and only those points are used for single-ellipse (blue), two-ellipse (green), and gaussian (red) model fits. Data from fewer eyes were available at extreme beam tilts (gray points). The peak eAC is at a negative beam tilt in the nasal retina, but a second peak is anticipated at high (roughly +20°) beam tilts, with an intervening “dip” around +10°. This is not well-captured by the single-ellipse model (blue), nor the gaussian model (red). For the nasal retina, the two-ellipse model starts with a large ellipse (semi-major axis 3.00, semi-minor axis 2.26; units in log-transformed m^-1^). From that ellipse, we subtract a small ellipse representing a “gap” in reflective microstructures, with a semi-major axis of 0.49 and a semi-minor axis of 0.10. Both ellipses have the same angle (+13°). The temporal retina has a similar fit (respective axes values: 2.99, 2.33, 0.52, 0.10) but angled in the opposite direction (−12°). Bottom: The data are re-plotted in polar-coordinates. Dashed green curves show the larger and smaller ellipses that make up the final two-ellipse model (solid line), while a dashed straight line shows the angle of the ellipses. The major axes for this two-ellipse model imply that microstructures in the inner retina are roughly aligned with the photoreceptors (whose long axes are depicted by orange hash marks in Fig 1C).

In contrast, representative sampling of the GCL (7%Depth; S2 Fig), the IPL (30%Depth; S3 Fig), INL (40%Depth; Fig 5), OPL (50%Depth; S4 Fig), and ONL (65%Depth; S5 Fig), revealed that neither hemiretina had the anticipated *tilt vs log(eAC)* relationship: It appeared that there were two separate peaks. Because this contour is poorly-characterized by either the gaussian or the single-ellipse model, we applied a two-ellipse model *post-hoc*: A large ellipse describes the tissue microstructures’ modulation of log(eAC) according to beam tilt *if there were no spacing/interruption in those microstructures.* A smaller ellipse is subtracted from the larger one. The smaller ellipse represents a translucent interruption in those microstructures. We constrained this two-ellipse model by requiring both ellipses to have the same angle of orientation, and for the smaller ellipse’s semi-minor axis to be log(eAC) = 0.1 m^-1^. Thus, only one new variable, the smaller ellipse’s semi-major axis (roughly, “What is the maximum light allowed through that interruption in the microstructures?”) is being added to the single-ellipse model. Because we are not aware of any pre-packaged algorithms for fitting this two-ellipse model precisely, random combinations of ellipses were tested until a good fit was generated. This was judged by minimization of the sum of squared errors (maximization of R^2^) in *tilt vs log(eAC)* space, with one caveat: For illustrative fits to group average data (Figs 5, S2–S5 Figs) we are interested in the layer-to-layer and nasal-versus-temporal consistency of a model outcomes. We therefore prevented two-ellipse model from “reducing” to the single-ellipse model nested inside, even if it would optimize R^2^ (e.g., S4 Fig) by obliging the semi-major axis of the smaller ellipse to have a log(eAC) > 0.3 m^-1^.

At the GCL, IPL, INL, OPL, and ONL, the two-ellipse models were separately fit to group average data from the temporal and nasal retinas. In all cases (Fig 5 and S2–S5 Figs) two-ellipse model fits were visibly satisfactory. Fits contrasted in an important way with the single-ellipse model: The single-ellipse implied that those microstructures were tilted +10° in the temporal retina – opposite of the photoreceptors (max eAC at tilt ≈ −10° at INT; Fig 4 and Table 1). The two-ellipse model instead described microstructure tilts of −12° to −16° in the temporal retina, implying that inner retinal microstructures are reasonably well-aligned with the photoreceptors. Estimates for the smaller ellipse’s semi-major axis were typically ~0.5 in the temporal retina. When compared to estimates of the large ellipse’s semi-major axis, (2.7 to 3.3), mindful that these numbers represent log-transformed coefficients, we extrapolate that (10^0.5^/10^3^≈)0.3% of the tissue is composed of “translucent interruptions”. Parallel findings in the nasal retina showed microstructure tilts of +10° to +14°– now aligned with photoreceptors (Table 1) – with similar estimates for semi-major axes.

**Table 1:** Estimated microstructure alignment in temporal and nasal retinal layers.

Layer	%Depth	Figure	Calculated Angle of Cellular Microstructures			
			Temporal Retina		Nasal Hemiretina	
			single-ellipse	two-ellipse	single-ellipse	two-ellipse
			mean ± s.e.m.	groupwise est.	mean ± s.e.m.	groupwise est.
**RNFL**	4	S1	+5.0 ± 1.1		+2.2 ± 2.6	
**GCL**	7	S2	+16.4 ± 6.3	−16	−20.9 ± 10.6	+10
**IPL**	30	S3	+14.8 ± 6.5	−13	−28.4 ± 7.2	+14
**INL**	40	5	+4.0 ± 5.2	−12	−29.2 ± 7.8	+13
**OPL**	50	S4	+18.6 ± 7.3	−14	−29.0 ± 6.9	+13
**ONL**	65	S5	+6.1 ± 8.1	−14	−15.9 ± 8.6	+10
**ELM**	75	S6	+3.5 ± 0.8		−0.8 ± 0.8	
**ELIP**	83.5	3	−8.8 ± 2.6		+5.2 ± 2.5	
*	86.5		−27.4 ± 4.7		+12.5 ± 1.4	
**INT**	92	S7	−13.0 ± 2.5		+8.2 ± 1.3	
**X**	94		−25.2 ± 4.9		+14.1 ± 1.9	

Layer * corresponds to the area of relatively low eACs between the ELIP and INT. Results from the single-ellipse model are also shown in Fig 4. The RNFL and ELM have angles numerically near zero (although temporal ELM is non-zero (q<0.05)), meaning they are most reflective when the beam is *roughly* perpendicular that retinal layer, as in Fig 1C. In layers containing rod inner and outer segments (ELIP, *, INT, and perhaps [16] layer X) microstructures have a negative angle, meaning they are most reflective (highest eAC) when the OCT beam is aimed temporal-to-nasal, as in Fig 1B. It is unclear which angle obtained – from ELIP, *, or INT – should be compared with histology, but they have the same valence, and their range includes literature measurements of photoreceptor tilt [13]. In the nasal retina, those same microstructures have a maximum eAC at a positive tilt, and are most reflective when the beam is aimed nasal-to-temporal (as in Fig 1D). For the rest of the retina (shaded values; GCL, IPL, INL, OPL, ONL), the single-ellipse model is inadequate compared to the two-ellipse model (Fig 5, S2–S5 Figs). The two-ellipse model argues that the microstructures in the GCL through ONL are well-aligned with the rod inner and outer segments: They have negative angles in the temporal retina, and positive ones in the nasal retina.

Finally, we explored fitting the two-ellipse model to data from individual eyes, instead of group averages as above. Binned data from each hemiretina, of each eye, were investigated at two places: The inner plexiform layer (30%Depth), which has the highest eAC (and so signal-to-noise) in the greyed area of Fig 4, and a “negative control” area of similar signal-to-noise but where the single-ellipse model was thought adequate, ELIP (83.5%Depth). The larger ellipse was allowed a semi-major axis between 2.0 m^-1^ and 4.0 m^-1^, and a semi-minor axis between 1.0 and 3.0 m^-1^ (covering the dynamic range for the neural retina, per Figs 2 and 4). The smaller ellipse again had an angle identical to the larger ellipse, a semi-minor axis of 0.1 m^-1^, and a semi-major axis between 0.1 m^-1^ and 1.0 m^-1^ (roughly double the estimates from groupwise data).

**Fig 6:**
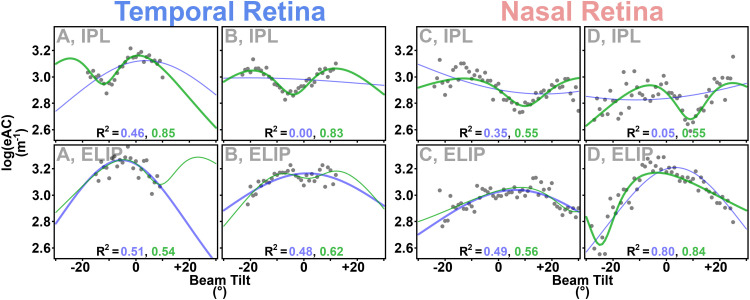
Data from four hemi-retinas (A, B, C, and D) illustrate log(eAC) variation with beam tilt at the inner plexiform layer (IPL, 30.0%Depth, top) and the ellipsoid zone (ELIP, 83.5%Depth, bottom). For IPL, eACs reach a local minimum at negative tilts in the temporal retina, and at positive tilts in the nasal retina – a pattern visible in group mean data (Table 1, S3 Fig). These tilt-specific “translucent interruptions” of diminished reflectivity are successfully modeled by subtracting a small ellipse from a large one: Here, the two-ellipse model (green line) is drawn thicker than the single-ellipse model (blue line) when it is superior (Bonferroni-corrected p < 0.05). Note that this pattern does not propagate to ELIP – as might happen if “shadowing” were the cause of low eACs in the more vitread IPL layer. In ELIP, the two-ellipse model can “slide” towards the most positive or negative tilts collected for a hemiretina (A, C, and D), thereby accommodating data that are reasonably well-modeled by the single-ellipse. This overfitting creates a challenge for unsupervised application of the two-ellipse model to individual hemiretinas. It also leads to inconsistency in the estimated tilt for the local minimum, as illustrated by C and D, which are respectfully from the right and left eye of the same animal: For ELIP, estimates are at +29° and −24°. In contrast, IPL estimates from C and D are consistent (both +10°).

In this *post-hoc* analysis at IPL, we compared the nested single-ellipse and two-ellipse models with an F-test: As with any multiple regression comparison of nested models, we tested whether the sum of squared errors (in *tilt vs log(eAC)* space) was significantly reduced by adding the additional parameter to make the two-ellipse model. We used a Bonferroni-corrected α = 0.05 to judge significance at each of the 54 hemiretinas. At IPL, the two-ellipse model was superior ~40% of the time (22 of 54). When the same analysis was repeated at ELIP, the two-ellipse model was superior in numerically fewer cases (16 of 54), and review of individual fits implied a qualitative difference between IPL and ELIP that is difficult to capture with this statistical approach (Fig 6).

## Discussion

In normal adult mice, we surveyed and modelled the influence of OCT beam tilt on the reflectivity of each retinal layer. The OCT appearance of *every* layer was at influenced by beam tilt (non-zero e″ in Fig 4). While this was expected in the RNFL and layers occupied by the photoreceptor inner and outer segments, it was somewhat surprising for the GCL through ONL [3,7,9]. Indeed, the influence of beam tilt was readily detectable in individual eyes: At every %Depth, at least half of the hemiretinas bore a significant relationship between tilt and log(eAC) (bottom of Fig 4). This has implications for future directional OCT (D-OCT) studies, making signal normalization of one retinal layer to another less appealing. Although beam tilt’s influence is small at the RPE (low-magnitude e″), in some applications even the RPE is a poor choice for signal normalization: Its reflectivity varies between mouse strains according to melanin content [9], and we consistently (Fig 2, top of Fig 4) found greater eACs in the nasal than in the temporal RPE – presumably due to the greater melanin content in the nasal than the temporal retina of C57 mice [22].

The retina is typically imaged with minimal tilt of the OCT beam. Even in control mice, this can generate solvable mysteries: ***(i)*** Why does reflectivity differ in the temporal versus nasal retina (Fig 2, middle panel)? Our single-ellipse model (Figs 3 and 4) summarizes the beam tilt eliciting peak reflectivity – intended to report on the preferential orientation of microstructures – and how reflective (maximum log(eAC)) or transparent (minimum log(eAC)) that part of the retina can be at a wide variety of beam tilts. From that model, we calculate that the orientation of most microstructures differs in the temporal versus nasal retina (Table 1, Fig 4) and after this is accounted for, we find only subtle regional differences in the neural retina (top two panels of Fig 4). These findings reasonably match literature expectations: Photoreceptors on each side of the optic nerve have opposite tilts relative to the retinal surface, but otherwise are grossly similar [13]. We calculate that photoreceptors are tilted by ~10° towards the pupil center, depending on which specific %Depth between ELIP and INT is used (Table 1) – an anatomic correlate of the Stiles-Crawford effect. ***(ii)*** In a second mystery, the identity of the hypodense band “X” is still debated. The present D-OCT data argue that microstructures in “X” and the photoreceptors (especially between ELIP and INT) are similarly tilted, and reflectivity is similarly tilt-dependent (e″ in Fig 4). This leads us to conclude that “X” is composed of photoreceptors, in agreement with observations of Bloom & Singal (in their terminology, band “3-4 HYPO”) indicating that “X” is part of the neural retina [16]. It appears to be the mouse homolog of the human “RPE-1” band, but inter-species comparisons require caution, as RPE-1’s visibility varies with proximity to the fovea [23]. Large dynamic changes in the size of “X” during light and dark-adaptation in mouse retina [24] further argues that “X” is not part of the RPE.

Prior work uses a gaussian model to describe the tilt-dependence of retinal reflectivity at the RNFL, ELM, and more-exterior layers [7,9]. In those retinal layers, we find that the gaussian model and the single-ellipse model are statistically non-inferior to one-another. Extrapolating the gaussian model to extreme beam tilts is problematic (Fig 3), and its greater number of parameters slightly hinders statistical power. On the other hand, it sometimes provides more accurate point estimates of reflectivity than the single-ellipse model (S6 Fig). For the aforementioned retinal layers [9], at clinically-useful beam tilts [12], our findings support continued use of either the gaussian or the single-ellipse model, depending on a project’s needs.

Our selection of ellipses to model D-OCT data was inspired by analysis methods for diffusion tensor MRI (see O’Donnell & Westin [25] for a brief review). In both imaging modalities, multiple grayscale images are acquired with a direction-sensitive variable (beam tilt for OCT, diffusion gradient for MRI), and direction-dependence is summarized to support inferences about microstructure alignment. Brain diffusion MRI is more mature, offering axon fiber tractography [26], the means of measuring cellularity of neuronal tissue during inflammation [27], and estimates other tissue characteristics that may be important in disease, like neurite density [28]. D-OCT may be able to adapt those analytic techniques: Our *post hoc* two-ellipse model is an adaptation of a diffusion MRI method of including a second ellipsoid to describe more than one cellular compartment [29].

Much of the retina – spanning the GCL through the ONL – was challenging to characterize with our pre-planned analyses with the gaussian and single-ellipse models. Still, reflectivity was tilt-dependent in most hemiretinas (bottom of Fig 4). The mathematical model used to characterize the influence of beam tilt is critical to the interpretation: The pre-planned single-ellipse model would have us search for an anatomical correlate angled ~30° relative to the photoreceptors (Table 1), and we know of no such structure. We favor the *post-hoc* two-ellipse model for this span of the retina, although application to individual hemiretinas is challenging (Fig 6): Often enough, it numerically superior to other models (Fig 5, S3 Fig), and it seemed to apply more consistently to group-average data than the gaussian model, which twice (Fig 3, S2 Fig) flipped upside down for one hemiretina to optimize fit. Moreover, the two-ellipse model argues that reflective microstructures are aligned roughly parallel to photoreceptor inner and outer segments (Table 1), which seems to better match the organization of the retina in published micrographs [30,31]. It does volunteer an interesting idea; that there are “translucent interruptions” to those microstructures. Macroscopic analogies might be deck prisms in a sailboat, or glass rods stuck into a cake.

The structural correlate of those “translucent interruptions” must span several neuronal layers, and both vascular and avascular territories of the retina (Fig 5, S2–S5 Figs). The location and optical properties implicate Müller glia, which are known to be low-scattering conduits for light [32,33]. Depending on the retinal layer and temporal/nasal hemiretina, the two-ellipse model predicts that Müller glia are tilted by ~±13°, in rough alignment with the photoreceptor inner and outer segments (Table 1). We are not aware of prior studies quantifying the tilt of Müller glia *per se*. However, the columnar organization of the ONL is parallel to the rod inner and outer segments in normal mice [13], and published micrographs labeled for glia-specific proteins show that Müller glia are tilted relative to the retina-vitreous border [30,31], and radial processes need not be parallel to microvilli [34,35] (the later are located at the ELM). Are Müller glia numerous-enough to explain why ≥ 0.3% of the retina surface area would be occupied by “translucent interruptions” between the RNFL and ELM? The lower bound of area occupied by Müller glia can be estimated with the cross-sectional area of the single, long, radially-oriented Müller glial process that spans from the RNFL to the ELM. In the mouse, its radius is ~ 1 µm [36]. Histological estimates are challenging because glial volumes are exquisitely sensitive to toxic and osmotic challenges [37], with Chao et al [36] estimating a shrinkage factor of 42%. Still, because a flat mount of the mouse retina contains 15,000 Müller glia [35] per mm^2^, those radially-oriented processes cumulatively occupy at least 0.047% of that area. Confocal images of the IPL published by Franze et al [32], imply that ~4% of the area is occupied by Müller glia. In short, literature-derived predictions for the size of a “translucent interruption” in the microstructures caused by Müller glia span two orders of magnitude (0.047% to 4%), bookending our OCT-derived value (≥0.3%). Future studies of light-dependent changes in the inner retina [4], or modulation of glia-specific aquaporins [38], may further characterize this putative OCT marker for Müller glia.

## Conclusion

Qualitatively, D-OCT is abnormal in dozens of ophthalmological diseases [39]. A quantitative approach to D-OCT may improve early detection and treatment monitoring for those disease. Planning such experiments requires a detailed understanding D-OCT findings for the normal healthy retina: We advanced that broader effort, clarifying reasons for nasal versus temporal differences in a standard OCT acquisition, highlighting the challenges of applying prior models to D-OCT measurements of several retinal layers, and identifying tilt-dependence in all retinal layers, which obliges caution when normalizing one layer to another. With the two-ellipse model, it may be possible to study Müller glia with D-OCT.

## Supporting information

S1 Fig Binned group-average data from the retinal nerve fiber layer (4%Depth) illustrate estimated attenuation coefficient (eAC) variation according to beam tilt. The temporal (figure left) and nasal (figure right) retina are analyzed separately. *Top:* Group mean (±s.e.m.) eACs as a function of beam tilt, formatted like in prior work [9]. Black points are used when at least nine eyes contribute to that mean, and only those points are used for single-ellipse (blue) and gaussian (red) model fits. Data from fewer eyes were available at extreme beam tilts (gray points). The peak eAC is at a slightly positive beam tilt in the temporal retina – indicating that this part of the retina is most reflective when the OCT beam starts nasal to the optic nerve head, and is aimed temporally (as in Fig 1, top). *Bottom:* The data are re-plotted in polar-coordinates.
(PDF)

S2 Fig Binned group-average data from the retina ganglion cell layer (7%Depth) illustrate eAC variation according to beam tilt. Data are displayed as in S1 Fig, save for the two-ellipse model fits (green). Relatively high eACs were measured at a negative beam tilt in the nasal retina, but a second peak is anticipated at high (>+20°) beam tilts, with an intervening “dip” around +12°. This is not well-captured by the single-ellipse (blue) and gaussian (red) models. For the nasal retina, the two-ellipse model starts with an ellipse with a semi-major axis of 3.14 (owing to log-transformation, this would be an eAC of 10^(3.14)^ m^-1^) and a semi-minor axis of 2.57 (10^(2.57)^ m^-1^). From that ellipse, we subtract a small ellipse representing a “gap” in reflective microstructures, with a semi-major axis of 0.39 and a semi-minor axis of 0.10. Both ellipses have the same angle (+10°). The temporal retina has a similar fit (respective axes values: 3.21, 2.68, 0.50, 0.10) but angled in the opposite direction (−16°). *Bottom:* The data are re-plotted in polar-coordinates. Dashed green curves show the larger and smaller ellipses that make up the final two-ellipse model (solid line), while a dashed straight line shows the angle of the ellipses.
(PDF)

S3 Fig Binned group-average data from the inner plexiform layer (30%Depth) illustrate eAC variation according to beam tilt. Data are displayed as in S2 Fig. The peak eAC is at a negative beam tilt in the nasal retina, but a second peak is anticipated at high (>+20°) beam tilts, with an intervening “dip” around +14°. This is not well-captured by the single-ellipse (blue) and gaussian (red) models. For the nasal retina, the two-ellipse model starts with an ellipse with a semi-major axis of 3.32 and a semi-minor axis of 2.65. From that ellipse, we subtract a small ellipse representing a “gap” in reflective microstructures, with a semi-major axis of 0.50 and a semi-minor axis of 0.10. Both ellipses have the same angle (+14°). The temporal retina has a similar fit (respective axes values: 3.34, 2.68, 0.51, 0.10) but angled in the opposite direction (−13°). *Bottom:* The data are re-plotted in polar-coordinates. Dashed green curves show the larger and smaller ellipses that make up the final two-ellipse model (solid line), while a dashed straight line shows the angle of the ellipses.
(PDF)

S4 Fig Binned group-average data from the outer plexiform layer (50%Depth) illustrate eAC variation according to beam tilt. Data are displayed as in S2 Fig. The peak eAC is at a negative beam tilt in the nasal retina, but a second local maximum is present at a tilt of roughly +18°. This is not well-captured by the single-ellipse (blue) and gaussian (red) models. For the nasal retina, the two-ellipse model starts with an ellipse with a semi-major axis of 3.29 and a semi-minor axis of 2.67. From that ellipse, we subtract a small ellipse representing a “gap” in reflective microstructures, with a semi-major axis of 0.46 and a semi-minor axis of 0.10. Both ellipses have the same angle (+13°). The temporal retina has a similar fit (respective axes values: 3.29, 2.78, 0.51, 0.10) but angled in the opposite direction (−14°). *Bottom:* The data are re-plotted in polar-coordinates. Dashed green curves show the larger and smaller ellipses that make up the final two-ellipse model (solid line), while a dashed straight line shows the angle of the ellipses.
(PDF)

S5 Fig Binned group-average data from the outer nuclear layer (65%Depth) illustrate eAC variation according to beam tilt. Data are displayed as in S2 Fig. The peak eAC is at a negative beam tilt in the nasal retina, but a second local maximum is present at a tilt of roughly +18°. This is not well-captured by the single-ellipse (blue) and gaussian (red) models. For the nasal retina, the two-ellipse model starts with an ellipse with a semi-major axis of 2.85 and a semi-minor axis of 2.11. From that ellipse, we subtract a small ellipse representing a “gap” in reflective microstructures, with a semi-major axis of 0.43 and a semi-minor axis of 0.10. Both ellipses have the same angle (+10°). The temporal retina has a grossly similar fit (respective axes values: 2.69, 2.50, 0.37, 0.10) but angled in the opposite direction (−14°). *Bottom:* The data are re-plotted in polar-coordinates. Dashed green curves show the larger and smaller ellipses that make up the final two-ellipse model (solid line), while a dashed straight line shows the angle of the ellipses.
(PDF)

S6 Fig Binned group-average data from the external limiting membrane (75%Depth) illustrate eAC variation according to beam tilt. Data are displayed as in S1 Fig. At this %Depth, the gaussian (red) model outperforms the single-ellipse (blue) model for tilts between −20° and +20°. Still, (***i***) both models adequately communicate that eAC is very sensitive to beam tilt in the measured range, (***ii***) both models adequately communicate that the peak eAC is at a tilt near 0°, and (***iii***) gaussian model predictions are implausible for tilts near 180° (Figure Bottom). Adding another predictor (a degree of freedom) to each model would improve fits. Whereas ellipses are subtracted to improve model fits in S2–S5 Figs, an additive two-ellipse model may be useful here. *Bottom:* The data are re-plotted in polar-coordinates.
(PDF)

S7 Fig Binned group-average data from the interdigitation layer (“INT”; 92%Depth) – sometimes referred to as the location of the outer segment (OS) tips – illustrate eAC variation according to beam tilt. Data are displayed as in S1 Fig. At this %Depth, the gaussian (red) and single-ellipse (blue) perform well-enough in the measured range of beam tilts that there was no need to fit a two-ellipse mode. *Bottom:* The data are re-plotted in polar-coordinates. As elsewhere, gaussian model predictions are implausible for tilts near 180°.
(PDF)

S8 Fig Binned group-average data from the retinal pigment epithelium (97%Depth) illustrate eAC variation according to beam tilt. Data are displayed as in S1 Fig. At this %Depth, the gaussian (red) and single-ellipse (blue) perform well-enough in the measured range of beam tilts that there was no need to fit a two-ellipse mode. *Bottom:* The data are re-plotted in polar-coordinates. As elsewhere, gaussian model predictions are implausible for the nasal retina at tilts near 180°.
(PDF)
