# Supplementary material for: Quantifying the influence of optical coherence tomography beam tilt in each retinal layer
Source: PLoS One. 2025 Jun 10;20(6):e0325217. doi: 10.1371/journal.pone.0325217 (PMC12186825; doi:10.1371/journal.pone.0325217)

S1 Fig. Binned group-average data from the retinal nerve fiber layer (4%Depth) illustrate estimated attenuation coefficient (eAC) variation according to beam tilt. The temporal (figure left) and nasal (figure right) retina are analyzed separately. _Top:_ Group mean (±s.e.m.) eACs as a function of beam tilt, formatted like in prior work [9]. Black points are used when at least nine eyes contribute to that mean, and only those points are used for single-ellipse (blue) and gaussian (red) model fits. Data from fewer eyes were available at extreme beam tilts (gray points). The peak eAC is at a slightly positive beam tilt in the temporal retina – indicating that this part of the retina is most reflective when the OCT beam starts nasal to the optic nerve head, and is aimed temporally (as in Fig.1, top). _Bottom:_ The data are re-plotted in polar-coordinates.

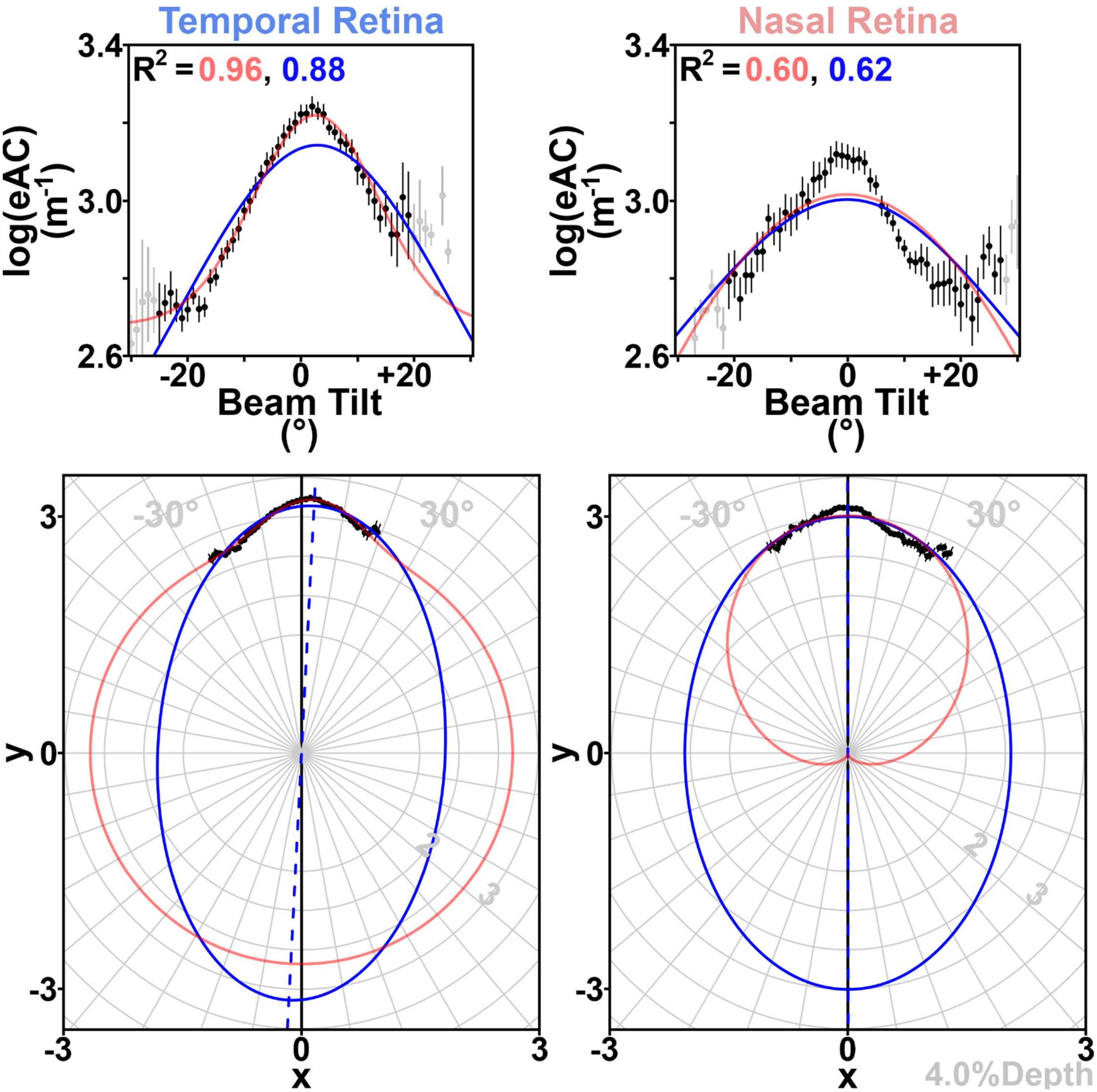

Supplement: S1 Fig — The temporal (figure left) and nasal (figure right) retina are analyzed separately. Top: Group mean (±s.e.m.) eACs as a function of beam tilt, formatted like in prior work [9]. Black points are used when at least nine eyes contribute to that mean, and only those points are used for single-ellipse (blue) and gaussian (red) model fits. Data from fewer eyes were available at extreme beam tilts (gray points). The peak eAC is at a slightly positive beam tilt in the temporal retina – indicating that this part of the retina is most reflective when the OCT beam starts nasal to the optic nerve head, and is aimed temporally (as in Fig 1, top). Bottom: The data are re-plotted in polar-coordinates. (PDF) [file pone.0325217.s001.pdf]
