# Supplementary material for: Quantifying the influence of optical coherence tomography beam tilt in each retinal layer
Source: PLoS One. 2025 Jun 10;20(6):e0325217. doi: 10.1371/journal.pone.0325217 (PMC12186825; doi:10.1371/journal.pone.0325217)

S2 Fig. Binned group-average data from the retina ganglion cell layer (7%Depth) illustrate eAC variation according to beam tilt. Data are displayed as in Figure S1, save for the two-ellipse model fits (green). Relatively high eACs were measured at a negative beam tilt in the nasal retina, but a second peak is anticipated at high (>+20°) beam tilts, with an intervening "dip" around +12°. This is not well-captured by the single-ellipse (blue) and gaussian (red) models. For the nasal retina, the two-ellipse model starts with an ellipse with a semi-major axis of 3.14 (owing to log-transformation, this would be an eAC of $10^{(3.14)}$ m^{-1}) and a semi-minor axis of 2.57 ($10^{(2.57)}$ m^{-1}). From that ellipse, we subtract a small ellipse representing a "gap" in reflective microstructures, with a semi-major axis of 0.39 and a semi-minor axis of 0.10. Both ellipses have the same angle (+10°). The temporal retina has a similar fit (respective axes values: 3.21, 2.68, 0.50, 0.10) but angled in the opposite direction (-16°). _Bottom:_ The data are re-plotted in polar-coordinates. Dashed green curves show the larger and smaller ellipses that make up the final two-ellipse model (solid line), while a dashed straight line shows the angle of the ellipses.

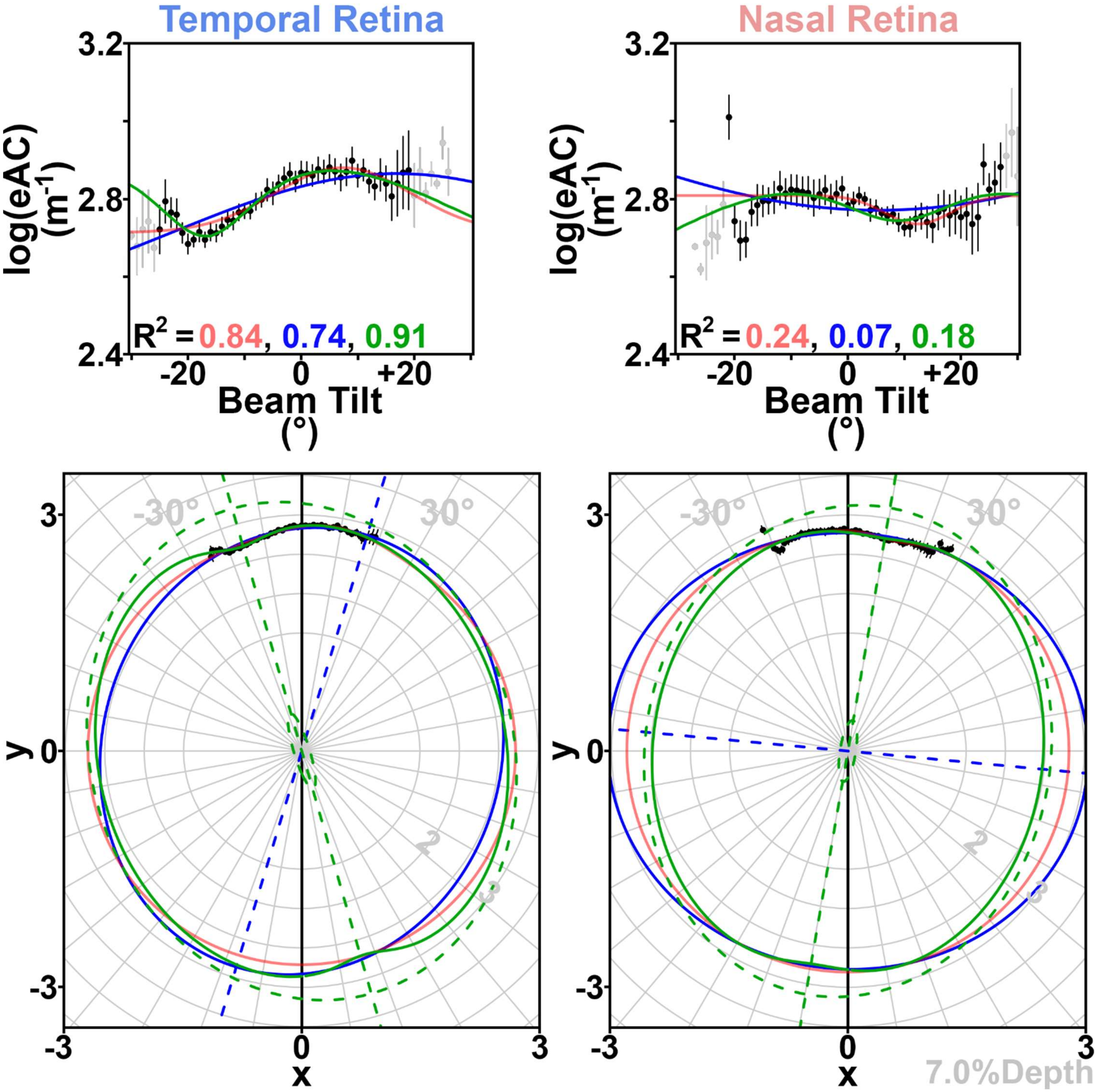

Supplement: S2 Fig — Data are displayed as in S1 Fig, save for the two-ellipse model fits (green). Relatively high eACs were measured at a negative beam tilt in the nasal retina, but a second peak is anticipated at high (>+20°) beam tilts, with an intervening “dip” around +12°. This is not well-captured by the single-ellipse (blue) and gaussian (red) models. For the nasal retina, the two-ellipse model starts with an ellipse with a semi-major axis of 3.14 (owing to log-transformation, this would be an eAC of 10(3.14) m-1) and a semi-minor axis of 2.57 (10(2.57) m-1). From that ellipse, we subtract a small ellipse representing a “gap” in reflective microstructures, with a semi-major axis of 0.39 and a semi-minor axis of 0.10. Both ellipses have the same angle (+10°). The temporal retina has a similar fit (respective axes values: 3.21, 2.68, 0.50, 0.10) but angled in the opposite direction (−16°). Bottom: The data are re-plotted in polar-coordinates. Dashed green curves show the larger and smaller ellipses that make up the final two-ellipse model (solid line), while a dashed straight line shows the angle of the ellipses. (PDF) [file pone.0325217.s002.pdf]
