# Supplementary material for: Quantifying the influence of optical coherence tomography beam tilt in each retinal layer
Source: PLoS One. 2025 Jun 10;20(6):e0325217. doi: 10.1371/journal.pone.0325217 (PMC12186825; doi:10.1371/journal.pone.0325217)

S4 Fig. Binned group-average data from the outer plexiform layer (50%Depth) illustrate eAC variation according to beam tilt. Data are displayed as in Fig S2. The peak eAC is at a negative beam tilt in the nasal retina, but a second local maximum is present at a tilt of roughly +18°. This is not well-captured by the single-ellipse (blue) and gaussian (red) models. For the nasal retina, the two-ellipse model starts with an ellipse with a semi-major axis of 3.29 and a semi-minor axis of 2.67. From that ellipse, we subtract a small ellipse representing a “gap” in reflective microstructures, with a semi-major axis of 0.46 and a semi-minor axis of 0.10. Both ellipses have the same angle (+13°). The temporal retina has a similar fit (respective axes values: 3.29, 2.78, 0.51, 0.10) but angled in the opposite direction (-14°). _Bottom:_ The data are re-plotted in polar-coordinates. Dashed green curves show the larger and smaller ellipses that make up the final two-ellipse model (solid line), while a dashed straight line shows the angle of the ellipses.

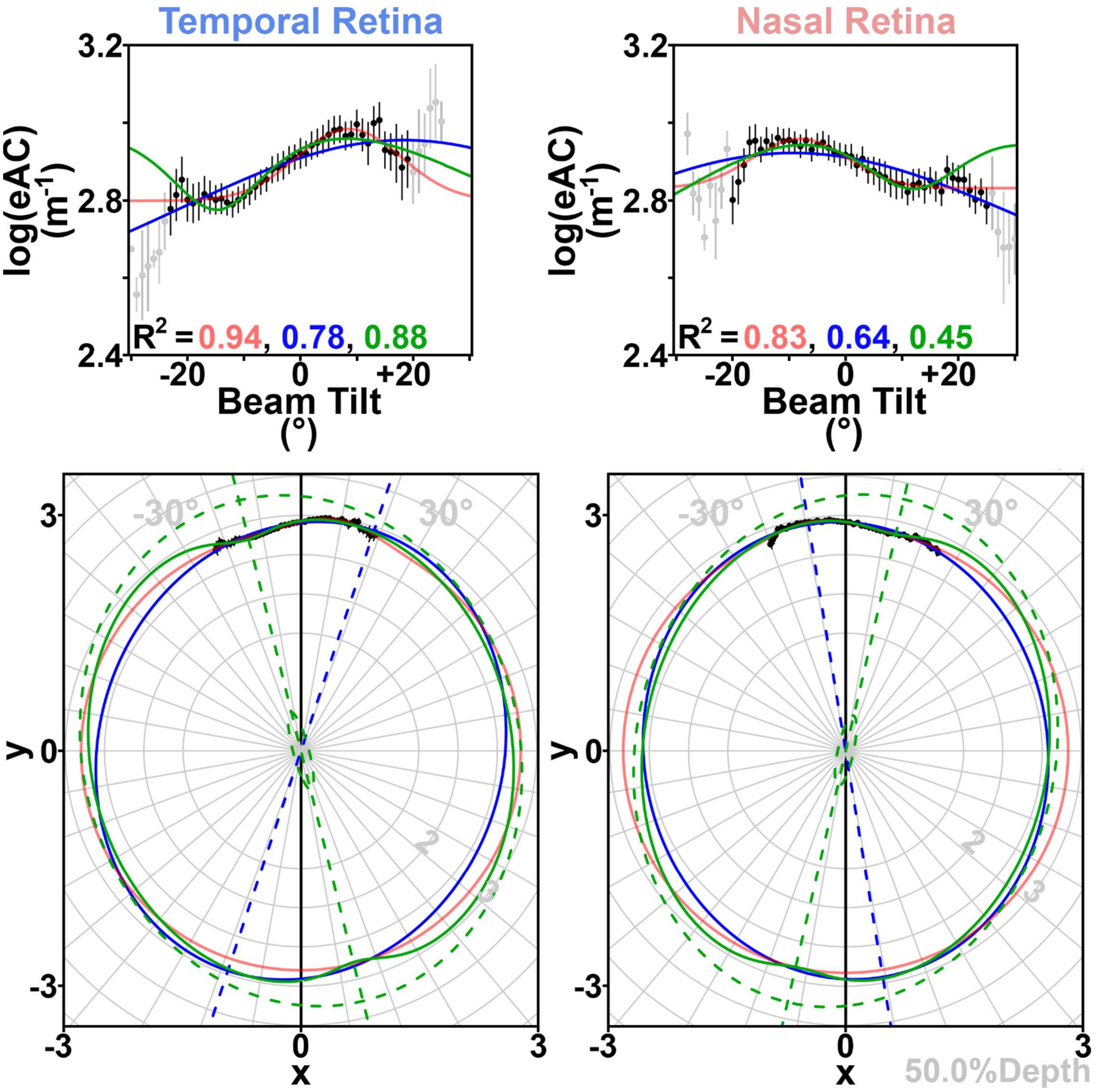

Supplement: S4 Fig — Data are displayed as in S2 Fig. The peak eAC is at a negative beam tilt in the nasal retina, but a second local maximum is present at a tilt of roughly +18°. This is not well-captured by the single-ellipse (blue) and gaussian (red) models. For the nasal retina, the two-ellipse model starts with an ellipse with a semi-major axis of 3.29 and a semi-minor axis of 2.67. From that ellipse, we subtract a small ellipse representing a “gap” in reflective microstructures, with a semi-major axis of 0.46 and a semi-minor axis of 0.10. Both ellipses have the same angle (+13°). The temporal retina has a similar fit (respective axes values: 3.29, 2.78, 0.51, 0.10) but angled in the opposite direction (−14°). Bottom: The data are re-plotted in polar-coordinates. Dashed green curves show the larger and smaller ellipses that make up the final two-ellipse model (solid line), while a dashed straight line shows the angle of the ellipses. (PDF) [file pone.0325217.s004.pdf]
