# Supplementary material for: Quantifying the influence of optical coherence tomography beam tilt in each retinal layer
Source: PLoS One. 2025 Jun 10;20(6):e0325217. doi: 10.1371/journal.pone.0325217 (PMC12186825; doi:10.1371/journal.pone.0325217)

S5 Fig. Binned group-average data from the outer nuclear layer (65%Depth) illustrate eAC variation according to beam tilt. Data are displayed as in Figure S2. The peak eAC is at a negative beam tilt in the nasal retina, but a second local maximum is present at a tilt of roughly +18°. This is not well-captured by the single-ellipse (blue) and gaussian (red) models. For the nasal retina, the two-ellipse model starts with an ellipse with a semi-major axis of 2.85 and a semi-minor axis of 2.11. From that ellipse, we subtract a small ellipse representing a “gap” in reflective microstructures, with a semi-major axis of 0.43 and a semi-minor axis of 0.10. Both ellipses have the same angle (+10°). The temporal retina has a grossly similar fit (respective axes values: 2.69, 2.50, 0.37, 0.10) but angled in the opposite direction (-14°). <u>*Bottom:*</u> The data are re-plotted in polar-coordinates. Dashed green curves show the larger and smaller ellipses that make up the final two-ellipse model (solid line), while a dashed straight line shows the angle of the ellipses.

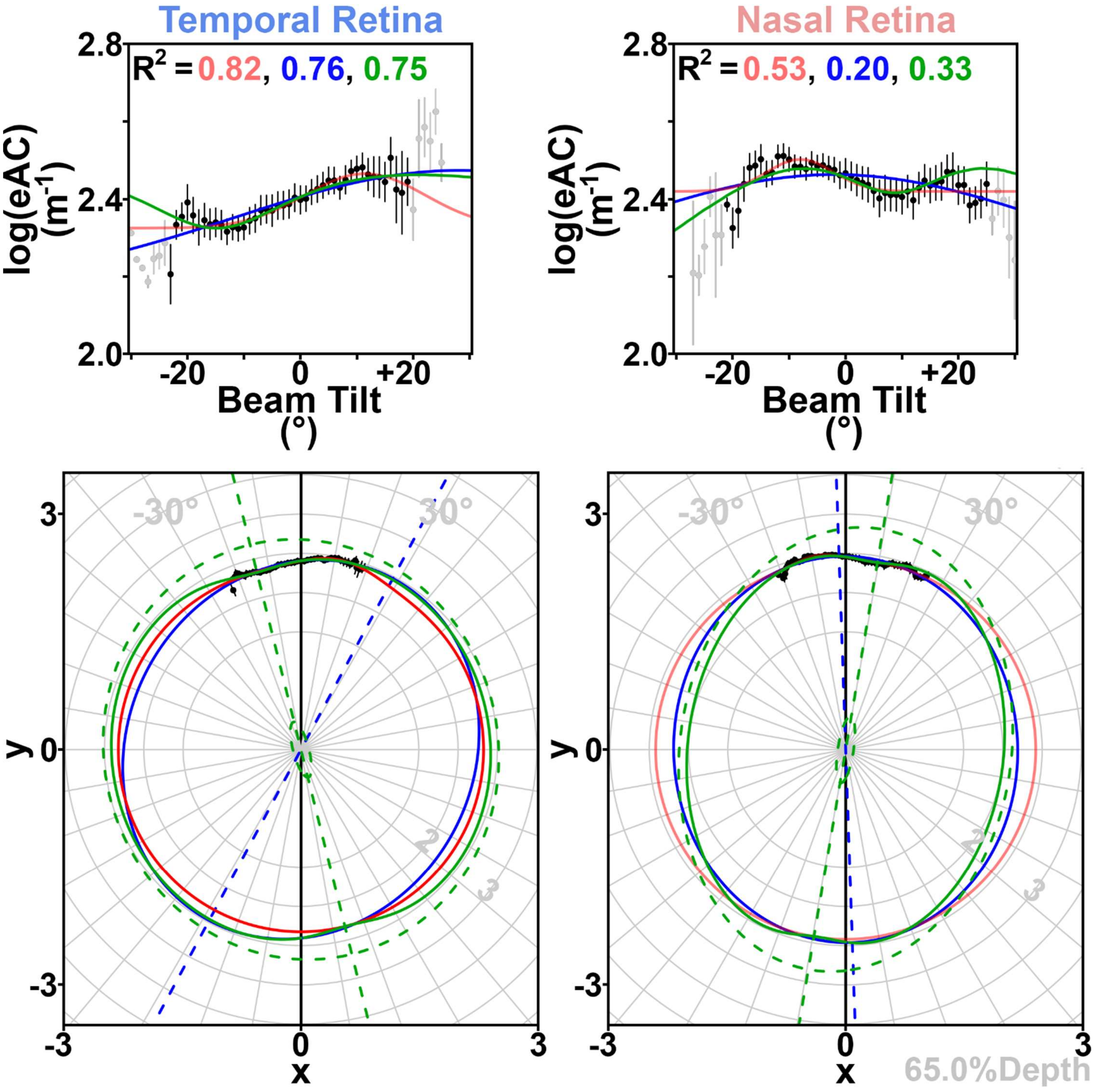

Supplement: S5 Fig — Data are displayed as in S2 Fig. The peak eAC is at a negative beam tilt in the nasal retina, but a second local maximum is present at a tilt of roughly +18°. This is not well-captured by the single-ellipse (blue) and gaussian (red) models. For the nasal retina, the two-ellipse model starts with an ellipse with a semi-major axis of 2.85 and a semi-minor axis of 2.11. From that ellipse, we subtract a small ellipse representing a “gap” in reflective microstructures, with a semi-major axis of 0.43 and a semi-minor axis of 0.10. Both ellipses have the same angle (+10°). The temporal retina has a grossly similar fit (respective axes values: 2.69, 2.50, 0.37, 0.10) but angled in the opposite direction (−14°). Bottom: The data are re-plotted in polar-coordinates. Dashed green curves show the larger and smaller ellipses that make up the final two-ellipse model (solid line), while a dashed straight line shows the angle of the ellipses. (PDF) [file pone.0325217.s005.pdf]
