# Supplementary material for: Quantifying the influence of optical coherence tomography beam tilt in each retinal layer
Source: PLoS One. 2025 Jun 10;20(6):e0325217. doi: 10.1371/journal.pone.0325217 (PMC12186825; doi:10.1371/journal.pone.0325217)

S6 Fig. Binned group-average data from the external limiting membrane (75%Depth) illustrate eAC variation according to beam tilt. Data are displayed as in Figure S1. At this %Depth, the gaussian (red) model outperforms the single-ellipse (blue) model for tilts between -20° and +20°. Still, (***i***) both models adequately communicate that eAC is very sensitive to beam tilt in the measured range, (***ii***) both models adequately communicate that the peak eAC is at a tilt near 0°, and (***iii***) gaussian model predictions are implausible for tilts near 180° (Figure Bottom). Adding another predictor (a degree of freedom) to each model would improve fits. Whereas ellipses are subtracted to improve model fits in Figures S2-S5, an additive two-ellipse model may be useful here. <u>*Bottom:*</u> The data are re-plotted in polar-coordinates.

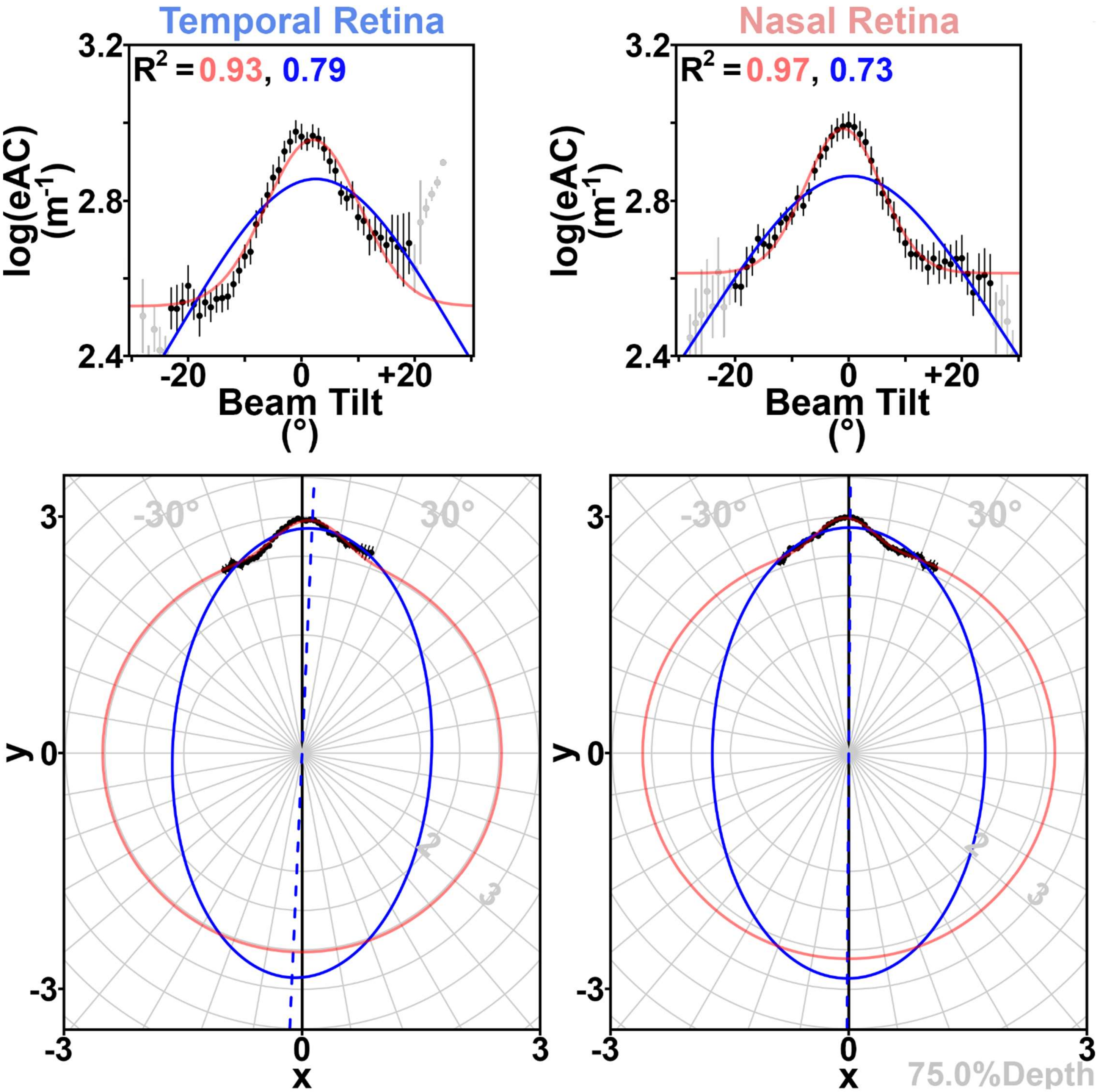

Supplement: S6 Fig — Data are displayed as in S1 Fig. At this %Depth, the gaussian (red) model outperforms the single-ellipse (blue) model for tilts between −20° and +20°. Still, (i) both models adequately communicate that eAC is very sensitive to beam tilt in the measured range, (ii) both models adequately communicate that the peak eAC is at a tilt near 0°, and (iii) gaussian model predictions are implausible for tilts near 180° (Figure Bottom). Adding another predictor (a degree of freedom) to each model would improve fits. Whereas ellipses are subtracted to improve model fits in S2–S5 Figs, an additive two-ellipse model may be useful here. Bottom: The data are re-plotted in polar-coordinates. (PDF) [file pone.0325217.s006.pdf]
