# Supplementary material for: Quantifying the influence of optical coherence tomography beam tilt in each retinal layer
Source: PLoS One. 2025 Jun 10;20(6):e0325217. doi: 10.1371/journal.pone.0325217 (PMC12186825; doi:10.1371/journal.pone.0325217)

S7 Fig. Binned group-average data from the interdigitation layer ("INT"; 92%Depth) – sometimes referred to as the location of the outer segment (OS) tips – illustrate eAC variation according to beam tilt. Data are displayed as in Figure S1. At this %Depth, the gaussian (red) and single-ellipse (blue) perform well-enough in the measured range of beam tilts that there was no need to fit a two-ellipse mode. _Bottom:_ The data are re-plotted in polar-coordinates. As elsewhere, gaussian model predictions are implausible for tilts near 180°.

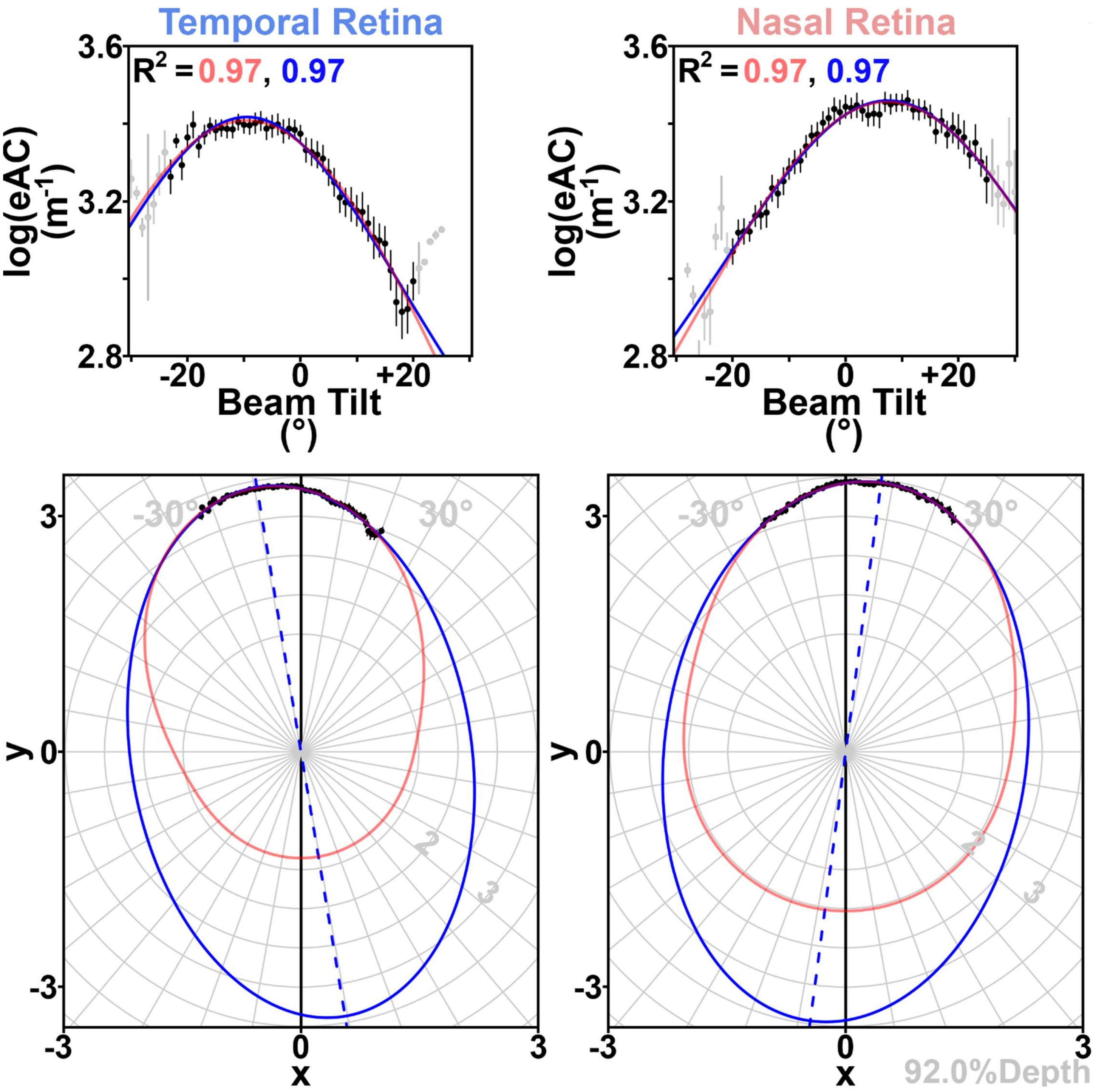

Supplement: S7 Fig — Data are displayed as in S1 Fig. At this %Depth, the gaussian (red) and single-ellipse (blue) perform well-enough in the measured range of beam tilts that there was no need to fit a two-ellipse mode. Bottom: The data are re-plotted in polar-coordinates. As elsewhere, gaussian model predictions are implausible for tilts near 180°. (PDF) [file pone.0325217.s007.pdf]
